# Accelerated maximum likelihood parameter estimation for stochastic biochemical systems

**DOI:** 10.1186/1471-2105-13-68

**Published:** 2012-05-01

**Authors:** Bernie J Daigle, Min K Roh, Linda R Petzold, Jarad Niemi

**Affiliations:** 1 Department of Computer Science, University of California Santa Barbara, California 93106, USA; 2 Department of Statistics, Iowa State University, Iowa 50011, USA

## Abstract

**Background:**

A prerequisite for the mechanistic simulation of a biochemical system is detailed knowledge of its kinetic parameters. Despite recent experimental advances, the estimation of unknown parameter values from observed data is still a bottleneck for obtaining accurate simulation results. Many methods exist for parameter estimation in deterministic biochemical systems; methods for discrete stochastic systems are less well developed. Given the probabilistic nature of stochastic biochemical models, a natural approach is to choose parameter values that maximize the probability of the observed data with respect to the unknown parameters, a.k.a. the maximum likelihood parameter estimates (MLEs). MLE computation for all but the simplest models requires the simulation of many system trajectories that are consistent with experimental data. For models with unknown parameters, this presents a computational challenge, as the generation of consistent trajectories can be an extremely rare occurrence.

**Results:**

We have developed Monte Carlo Expectation-Maximization with Modified Cross-Entropy Method (MCEM^2^): an accelerated method for calculating MLEs that combines advances in rare event simulation with a computationally efficient version of the Monte Carlo expectation-maximization (MCEM) algorithm. Our method requires no prior knowledge regarding parameter values, and it automatically provides a multivariate parameter uncertainty estimate. We applied the method to five stochastic systems of increasing complexity, progressing from an analytically tractable pure-birth model to a computationally demanding model of yeast-polarization. Our results demonstrate that MCEM^2^ substantially accelerates MLE computation on all tested models when compared to a stand-alone version of MCEM. Additionally, we show how our method identifies parameter values for certain classes of models more accurately than two recently proposed computationally efficient methods.

**Conclusions:**

This work provides a novel, accelerated version of a likelihood-based parameter estimation method that can be readily applied to stochastic biochemical systems. In addition, our results suggest opportunities for added efficiency improvements that will further enhance our ability to mechanistically simulate biological processes.

## Background

Conducting accurate mechanistic simulations of biochemical systems is a central task in computational systems biology. For systems where a detailed model is available, simulation results can be applied to a wide variety of tasks including sensitivity analysis, *in silico* experimentation, and efficient design of synthetic systems [1]. Unfortunately, mechanistic models for many biochemical systems are not known; consequently, a prerequisite for the simulation of these systems is the determination of model structure and kinetic parameters from experimental data.

Despite recent advances in experimental methodology, the estimation of unknown kinetic parameters from data is a bottleneck for performing accurate simulations [2]. For deterministic models of biochemical systems, where dynamics are typically described by ordinary differential equations, reliable methods for parameter estimation are relatively abundant [3]. In contrast, parameter estimation for stochastic biochemical systems are less well developed [4]. In recent years it has become increasingly clear that stochasticity plays a crucial role in many biological processes, ranging from bistable genetic switches [5-7] to robust oscillators [8,9]. Unlike in the deterministic regime, the dynamics of a stochastic system are described by a probability distribution which cannot usually be obtained analytically (although approximate methods such as finite state projection have been used with some success [10]). Instead, sampling methods like the stochastic simulation algorithm (SSA) [11] are used to generate ensembles of trajectories from the unknown distribution.

Given the probabilistic nature of stochastic biochemical models, a natural approach for parameter estimation is to choose values that maximize the probability of the observed data with respect to the unknown parameters (maximum likelihood estimates or MLEs). In the case of fully observed data, where the number of molecules of each system species is known at all time points, MLEs can be calculated analytically. However, since realistic biochemical systems are discretely and partially observed, computational MLE methods are necessary. One of the earliest examples presented, simulated maximum likelihood (SML), combines a non-parametric density function estimator with Monte Carlo simulation to approximate the likelihood function [12]. To maximize the likelihood, SML uses a genetic algorithm requiring absolute bounds on each of the unknown parameters. Horváth and Manini developed an expectation-maximization (EM) approach (see Methods) which artificially modifies a subset of reactions in simulated trajectories to approximate and maximize the likelihood [13]. However, this method can become increasingly inaccurate as species counts approach zero, and it is not clear how to properly choose the number of reactions to modify at each step. More recently, a histogram-based Monte Carlo simulation procedure was developed to estimate data likelihood [2]. Like the SML method, this approach uses a genetic algorithm to maximize the likelihood, requiring prior parameter bounds. Finally, Wang *et al.* proposed a method combining stochastic gradient descent (SGD) with a reversible jump Markov chain Monte Carlo sampler to maximize parameter likelihood [4]. The SGD method efficiently and heuristically generates trajectories consistent with observed data, iteratively modifying them via a Metropolis-Hastings step until they closely approximate trajectories from the unknown probability distribution.

Although not strictly an MLE method, Boys *et al.* developed a Bayesian approach for inferring parameters that employs a Poisson process approximation to efficiently generate trajectories consistent with observed data [14]. Like SGD, this method also incorporates a Metropolis-Hastings sampling step to correct for the approximate nature of the generated trajectories.

All of the above MLE approaches essentially iterate between two steps: (A) approximating a parameter likelihood using Monte Carlo sampling and (B) maximizing that approximation with respect to the unknown parameters using an optimization algorithm. We note that the Bayesian method of Boys *et al.* also requires extensive Monte Carlo sampling in the manner of step (A). Execution of (A) requires the generation of many system trajectories that are consistent with experimental data. When simulating trajectories of a model with unknown parameters, the generation of even a single trajectory consistent with data can be an extremely rare occurrence. The SML and histogram-based methods [2,12] mitigate this computational challenge by requiring accurate bounds for each unknown parameter. In contrast, the EM-based, SGD, and Poisson approximation methods [4,13,14] reduce simulation cost by generating system trajectories in a heuristic manner. Although these strategies have been successful, parameter bounds are not always available, and it is not clear whether heuristically generated trajectories can be used to accurately and efficiently parameterize all systems. In addition, unlike Bayesian methods, existing MLE approaches only return parameter point estimates without quantifying estimation uncertainty.

In this work, we develop Monte Carlo Expectation-Maximization with Modified Cross-Entropy Method (MCEM^2^), a novel, accelerated approach for computing MLEs along with uncertainty estimates. MCEM^2^ combines advances in rare event simulation [15-18] with an efficient version of the Monte Carlo EM (MCEM) algorithm [19], and it does not require prior bounds on parameters. Unlike the EM-based, SGD, and Poisson approximation methods above, MCEM^2^ generates probabilistically coherent system trajectories using the SSA. The remainder of the paper is structured as follows: We first provide derivation and implementation details of MCEM^2^ (Methods). Next, we apply our method to five stochastic biochemical models of increasing complexity and realism: a pure-birth process, a birth-death process, a decay-dimerization, a prokaryotic auto-regulatory gene network, and a model of yeast-polarization (Results). Through these examples, we demonstrate the superior performance of MCEM^2^ to an existing implementation of MCEM and the SGD and Poisson approximation methods. Finally, we discuss the distinguishing features of our method and motivate several promising future areas of research (Discussion).

## Methods

### Discrete-state stochastic chemical kinetic system

We focus on stochastic biochemical models that assume a well-stirred chemical system with *N* species {*S*_1_,…,*S*_*N*_}, whose discrete-valued molecular population numbers evolve through the firing of *M* reactions {*R*_1_,…,*R*_*M*_}. We represent the state of the system at time *t* by the *N*-dimensional random process **X**(*t*)≡(*X*_1_(*t*),…,*X*_*N*_(*t*)), where *X*_*i*_(*t*) corresponds to the number of molecules of *S*_*i*_ at time *t*. Associated with each reaction is its propensity function *a*_*j*_(**x**)(*j*=1,…,*M*), whose product with an infinitesimal time increment d*t* gives the probability that reaction *R*_*j*_ fires in the interval *tt* + d*t*) given **X**(*t*)=**x**. The sum of all *M* propensity functions for a given system state **x** is denoted *a*_0_(**x**). We restrict our attention to reactions that obey mass action kinetics—i.e. where *a*_*j*_(**x**)≡*θ*_*j*_*h*_*j*_(**x**) with *θ*_*j*_ a positive real kinetic constant and *h*_*j*_(**x**) a function that quantifies the number of possible ways reaction *R*_*j*_ can occur given system state **x**. Examples of *h*_*j*_(**x**) include: 1, *x*_1_$\frac{1}{2}x_{1}(x_{1}-1)$, and *x*_1_*x*_2_ for zeroth-order, unimolecular, homo-bimolecular, and hetero-bimolecular reactions, respectively. Further details on mass action propensity functions can be found in [20].

The “direct method” implementation of Gillespie’s stochastic simulation algorithm (SSA) provides a simple numerical procedure for generating exact system trajectories from their underlying (intractable) probability distribution [11]. The method works by sequentially simulating the time to the next reaction (*τ*) as an exponential random variable with mean 1/*a*_0_(**x**) and the index of the next reaction (*j’*) as a categorical random variable with probabilities *a*_*j*_(**x**)/*a*_0_(**x**)(*j*=1,…,*M*). Given a final time *T* and initial system state **X**(0)=**x**_0_, application of the direct method yields a reaction trajectory **z**≡(*τ*_1_*j*_1_^*′*^,…,*τ*_*r*_*j*_*r*_^*′*^), where *r* is the total number of reactions that happen to fire by time *T*. Although **z** is only of length *2r*, combining it with **x**_0 _ allows us to identify the complete system state at any time in the interval [0,*T* regardless of how large *N* and *M* are. Using the above notation, we can express the likelihood of the complete system trajectory (**x**_0_**z**) as the following function of the kinetic parameters ***θ***≡(*θ*_1_,…,*θ*_*M*_) (see [21] for a detailed derivation):

(1) $$\begin{array}{ll} f_{\theta }(x_{0},z) & =\left(\overset{r}{\underset{i=1}{\prod }}\theta_{j_{i}^{\prime }}h_{j_{i}^{\prime }}(x_{i-1})\right)\\ & \;\times \exp\left(-\overset{r+1}{\underset{i=1}{\sum }}\left[\tau_{i}\overset{M}{\underset{j=1}{\sum }}\theta_{j}h_{j}(x_{i-1})\right]\right), \end{array}$$

where *τ*_*r* + 1 _ is the time interval between the firing of the final reaction and *T*, and **x**_*i*−1 _ is the easily computable system state at the time immediately after the (*i*−1)^st^ firing event (i.e. when $t=\overset{i-1}{\underset{l=1}{\sum }}\tau_{l}$ for *i*>1).

### Maximum likelihood parameter estimation

If the true values of the kinetic parameters ***θ***^∗ ^ are unknown and we are given a complete system trajectory (**x**_0_**z**), a natural approach for generating parameter estimates $\hat{\theta }$ is to choose values of ***θ*** that maximize the likelihood with respect to the trajectory (Equation (1)). These maximum likelihood parameter estimates (MLEs) can be analytically computed for each reaction as follows (see [21] for a derivation):

(2) $${\hat{\theta }}_{j}=\frac{r_{j}}{\overset{r+1}{\underset{i=1}{\sum }}h_{j}(x_{i-1})\tau_{i}}.$$

where *r*_*j*_ is the total number of times reaction *R*_*j*_ fires in **z**. Although simple, Equation (2) is only useful in the presence of a complete system trajectory. Experimentally observed data are typically much less informative, consisting of the initial system state plus numbers of molecules for a subset of the system species at *d* discrete time points. We represent these “observed data” with **y**≡(**x**_0_**x**_1_^*′*^,…, **x**_*d*_^*′*^), where **x**_*i*_^*′*^ contains the numbers of molecules of a subset of the *N* species at some time point *t*_*i*_. Knowledge of any **y**of finite size is insufficient for reconstructing the complete system trajectory (**x**_0_**z**) and the corresponding likelihood (Equation (1)); thus, Equation (2) is not a feasible approach for computing MLEs. Instead, we require a method that can accommodate “unobserved data”—i.e., the states of all system species at all times not included in the observed data.

In this work we use the expectation-maximization (EM) algorithm [22] to identify MLEs in the presence of unobserved data. This algorithm suggests the following iterative computation given some ${\hat{\theta }}^{\left(0\right)}$ (see [23] for details):

(3) $$\begin{array}{ll} {\hat{\theta }}^{\left(n+1\right)} & =\underset{\theta }{\text{argmax}}Q(\theta |{\hat{\theta }}^{\left(n\right)})\\ & \equiv \underset{\theta }{\text{argmax}}\left(피\left[\log f_{\theta }(x_{0},z)|y,{\hat{\theta }}^{\left(n\right)}\right]\right)\\ & =\underset{\theta }{\text{argmax}}\left(\underset{z\in \theta (y)}{\sum }\left[g(z|y,{\hat{\theta }}^{\left(n\right)})\times \log f_{\theta }(x_{0},z)\right]\right), \end{array}$$

where 피·|y,θ^(n) is the expectation operator taken with respect to the conditional distribution of **z** given **y** and ${\hat{\theta }}^{\left(n\right)}$θ(y) is the set of all valid reaction trajectories that are consistent with **y**(i.e. trajectories that pass through all observed data points exactly), and $g(z|y,{\hat{\theta }}^{\left(n\right)})$ represents the unknown conditional density of **z**. The theory behind the EM algorithm guarantees that Equation (3) will converge to estimates that locally maximize the observed data likelihood, given *n* sufficiently large (Section 3 of [22]). Unfortunately, we cannot work with Equation (3) directly, as an explicit evaluation of the summation is intractable. Instead, we use a Monte Carlo extension of EM (MCEM) [24] that samples reaction trajectories using the direct method of the SSA to approximate ${\hat{\theta }}^{\left(n+1\right)}$:

(4) $$\begin{array}{ll} {\hat{\theta }}^{\left(n+1\right)} & \approx \underset{\theta }{\text{argmax}}\left(\overset{K}{\underset{k=1}{\sum }}\left[I\left(z_{k}^{\left(n\right)}\in 풵(y)\right)\right)\right)\\ & \times \left(\left(\log f_{\theta }\left(x_{0},z_{k}^{\left(n\right)}\right)\right]\right)\\ & =\underset{\theta }{\text{argmax}}\left(\overset{K^{\prime }}{\underset{k^{\prime }=1}{\sum }}\log f_{\theta }\left(x_{0},z_{k^{\prime }}^{\left(n\right)}\right)\right), \end{array}$$

where $z_{k}^{\left(n\right)}$ is the *k*^th^ SSA trajectory simulated using the parameter vector ${\hat{\theta }}^{\left(n\right)}$$I\left(z_{k}^{\left(n\right)}\in 풵(y)\right)$ is an indicator function taking a value of 1 if $z_{k}^{\left(n\right)}$ is consistent with **y**(and 0 otherwise), and *K* is the total number of simulated trajectories. Equation (4b) presents a simplified expression in which *k*^*′*^ indexes only the *K*^*′*^ simulated trajectories that are consistent with the observed data. In practice, we set *K* to the value that leads to the desired number of consistent trajectories *K*^*′*^. We note that Equations (4a) and (4b) describe a rejection sampling approach to generating reaction trajectories conditional to the observed data, in which only those simulated trajectories consistent with data are retained and all others are rejected. In practice, we simulate trajectories incrementally between two data points at a time, further propagating only those trajectories that pass through the second data point exactly. Although this incremental approach is much more efficient than performing rejection sampling across full length trajectories, as we describe below it can still be computationally prohibitive.

By simplifying Equation (4b) with the same procedure used to derive Equation (2) [21], we obtain an iterative, MCEM version of the MLE for each reaction:

(5) $${\hat{\theta }}_{j}^{\left(n+1\right)}=\frac{\overset{K^{\prime }}{\underset{k^{\prime }=1}{\sum }}r_{jk^{\prime }}^{\left(n\right)}}{\overset{K^{\prime }}{\underset{k^{\prime }=1}{\sum }}\left[\overset{r_{k^{\prime }}^{\left(n\right)}+1}{\underset{i=1}{\sum }}h_{j}(x_{i-1,k^{\prime }}^{\left(n\right)})\tau_{ik^{\prime }}^{\left(n\right)}\right]}.$$

Equation (5) is analogous to Equation (2), with trajectory features having an added subscript *k*^*′*^ and superscript (*n*).

An open question in the use of MCEM involves efficient selection of the numbers of consistent trajectories *K*^*′*^ and iterations *n*. We adopt the ascent-based MCEM algorithm [19] for this task, which suggests increasing *K*^*′*^ at each iteration according to an estimate of the current Monte Carlo error and terminating the algorithm when the estimated change in conditional log-likelihood $\left(피\left[\log f_{\theta }(x_{0},z)|y,{\hat{\theta }}^{\left(n\right)}\right]\right)$ passes below a constant threshold. Specifically, we set the initial value of *K*^*′*^ to 10 and the sample size increment parameters *αβ*, and *k* to their respective default values of .25, .25, and 3. We terminate the algorithm when an upper bound of the change in conditional log-likelihood (using *γ*=.25) was less than .005 for three consecutive iterations (see [19] for more details).

### Accelerating MLE computation

Equation (5) requires the generation of *K*^*′*^ trajectories that are consistent with observed data. For datasets with closely spaced time points and reasonably accurate initial parameter estimates ${\hat{\theta }}^{\left(0\right)}$, this task may be computationally feasible. For the more realistic case of a sparse dataset and inaccurate values of ${\hat{\theta }}^{\left(0\right)}$, it quickly becomes intractable, as the simulation of even one consistent trajectory is an extremely rare event. In light of this fact, we adapt methods from rare event simulation to substantially accelerate the use of MCEM. Below we describe the incorporation of three techniques: the cross-entropy method, multilevel splitting, and parameter perturbation. Specifically, we employ these three techniques together as a standalone algorithm to quickly compute plausible parameter estimates ${\hat{\theta }}^{\mathrm{CE}}$ (see below for details). We then use these parameter estimates as input to an otherwise unmodified ascent-based MCEM algorithm, which further refines the estimates until MLEs are obtained. The advantage of this two-step process is that we retain all of the desirable properties of MCEM while dramatically accelerating the time to convergence (due to the use of much more accurate MCEM initial parameter estimates).

#### The cross-entropy method

The cross-entropy (CE) method was first developed by Rubinstein [15] to accelerate the simulation of stochastic rare events. Since that time, the method has been used in many contexts, including combinatorial optimization [25] and stochastic biochemical modeling [18]. Briefly, the CE method begins by simulating *K* trajectories using an initial parameter vector ${\hat{\theta }}^{\left(0\right)}$. Next, a subset of ⌉*ρK*⌉ trajectories (with *ρ*∈[0,1] and ⌉·⌉ the ceiling function) that are closest to a given system state (i.e. observed data) is selected and used to compute a better parameter estimate ${\hat{\theta }}^{\left(1\right)}$. This process is then repeated until all ⌉*ρK*⌉ subset trajectories reach the given state, upon which the algorithm computes a final parameter vector ${\hat{\theta }}^{\mathrm{CE}}$ and terminates. Unless otherwise noted in the examples below, we set *K*=10^4^ and *ρ*=.001, which were shown empirically to confer good performance (see Discussion).

When applied to the task of stochastic parameter estimation, the CE method proposes an iterative optimization very similar to Equation (4a):

(6) $$\begin{array}{ll} {\hat{\theta }}^{\left(m+1\right)} & =\underset{\theta }{\text{argmax}}\left(\overset{K}{\underset{k=1}{\sum }}\left[I(d(z_{k}^{\left(m\right)},y)\leq \delta^{\left(m\right)})\right)\right)\\ & \;\left(\left(\times \log f_{\theta }(x_{0},z_{k}^{\left(m\right)})\right]\right) \end{array}$$

where $d(z_{k}^{\left(m\right)},y)$ is a user-defined function measuring the distance between a simulated trajectory and the observed data, and *δ*^(*m*)^ is the (*ρ*×100)^th ^ quantile of distances achieved by the *K* simulated trajectories. In this work, we choose *d*(·,·) to be a normalized *L*_1 _ distance evaluated at each observed time point for each observed species (i.e. we divide each absolute deviation by the quantity [1 + the value of the corresponding data point]). Upon simplification of Equation (6), we obtain the following expression for each CE reaction parameter:

(7) $${\hat{\theta }}_{j}^{\left(m+1\right)}=\frac{\overset{K}{\underset{k=1}{\sum }}\left[I(d(z_{k}^{\left(m\right)},y)\leq \delta^{\left(m\right)})\times r_{\mathrm{jk}}^{\left(m\right)}\right]}{\overset{K}{\underset{k=1}{\sum }}\left[I(d(z_{k}^{\left(m\right)},y)\leq \delta^{\left(m\right)})\times \overset{r_{k}^{\left(m\right)}+1}{\underset{i=1}{\sum }}h_{j}(x_{i-1,k}^{\left(m\right)})\tau_{\mathrm{ik}}^{\left(m\right)}\right]}.$$

Once *δ*^(*m*)^=0, Equation (7) is used a final time to obtain ${\hat{\theta }}^{\mathrm{CE}}$ and the algorithm terminates. If we then set ${\hat{\theta }}^{\left(0\right)}\equiv {\hat{\theta }}^{\mathrm{CE}}$ for MCEM, we expect that on average only *K*^*′*^/*ρ* total trajectories must be simulated to provide *K*^*′*^ consistent trajectories. Generally speaking, the algorithm is guaranteed to terminate provided *ρ* and *K*^*′*^ are sufficiently small and sufficiently large, respectively (see [26] and below for more details). As will be shown below, use of the CE method coupled with MCEM provides enormous computational savings when compared to MCEM initiated with arbitrary parameter values.

#### Multilevel splitting

If the observed data consist of many time points, simulating a trajectory that passes through all of the data will be extremely unlikely, even when using the true parameter values. Consequently, our CE method will require a very small *ρ* (with accompanying very large *K*) in order to converge in a reasonable number of iterations. As a means of reducing this computational expense, we have added a “divide and conquer” approach with respect to the data inspired by multilevel splitting (MS) methods for rare event simulation [16,17]. MS methods divide rare trajectories leading to a given system state into less rare sub-trajectories passing through intermediate states. Sub-trajectories that reach the intermediate states in a given time are split into multiple copies, while the others are killed with some probability. In this way, an ensemble of simulated trajectories is gradually enriched for those that reach the state of interest.

**Figure 1:**
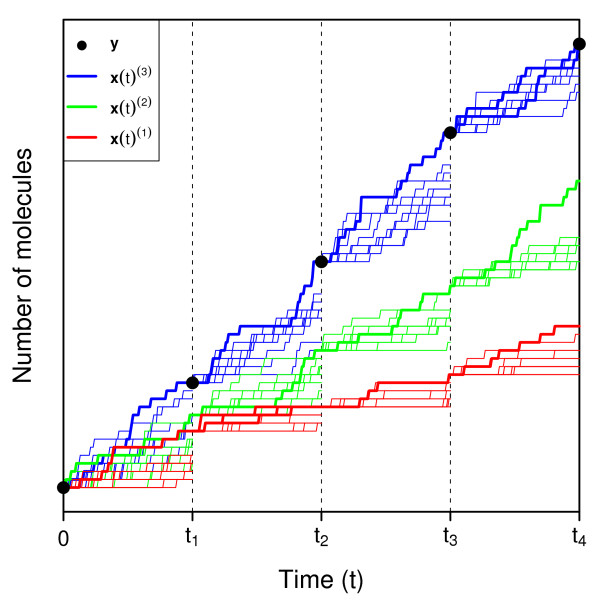
**Multilevel splitting applied to CE phase of MCEM**^**2**^**.** Using ${\hat{\theta }}^{\left(0\right)}$, we first simulate an ensemble of *K* trajectories from the initial system state (black circle at *t*=0) until time *t*_1 _ (red traces). The ending states of the ⌉*ρK*⌉ trajectories closest to the first observed data point (bold red traces) are sampled with replacement to provide starting states for the next simulation interval. We then simulate a second ensemble of *K* trajectories starting at time *t*_1 _ until reaching *t*_2_. Here, we select the ⌉*ρK*⌉ trajectories spanning the interval [0,*t*_2_] that are closest to the first and second data points (black circles at times *t*_1 _ and *t*_2_) and use them to initiate the third simulation ensemble. We repeat this process until reaching *t*_4_, at which time we compute the first set of parameter estimates ${\hat{\theta }}^{\left(1\right)}$ using the ⌉*ρK*⌉ trajectories closest to all data points (full length bold red traces). Using ${\hat{\theta }}^{\left(1\right)}$, we begin the process again at *t*=0, producing the green traces. Finally, using ${\hat{\theta }}^{\left(2\right)}$ to generate the blue traces, we obtain ⌉*ρK*⌉ trajectories coinciding exactly with all data points, which we use to compute ${\hat{\theta }}^{\mathrm{CE}}\equiv {\hat{\theta }}^{\left(3\right)}$.

A natural definition of a sub-trajectory in the context of observed data is the portion of a trajectory from time 0 to a recorded time point *t*_*i*_≤*t*_*d*_. Starting from *t*=0 for a given iteration of our CE method, we simulate *K* trajectories only until the first observed time point, giving rise to the sub-trajectories $\left(z_{1,1}^{\left(m\right)},z_{1,2}^{\left(m\right)},\ldots ,z_{1,K}^{\left(m\right)}\right)$, where the first subscript of $z_{i,k}^{\left(m\right)}$ denotes a sub-trajectory spanning the time interval [0,*t*_*i*_]. We then compute the distance $d(z_{1,k}^{\left(m\right)},y_{1})$ of each sub-trajectory with respect to the first observed data point **y**_1_≡(**x**_0_,**x**_1_^*′*^). Sub-trajectories falling in the (*ρ*^*′*^×100)^th^ quantile of distances (where we typically choose *ρ*^*′*^=*ρ*) are “split” by sampling from them with replacement to generate *K* new trajectories, while the remaining trajectories are killed. The new trajectories are simulated forward to the second observed time point to yield $\left(z_{2,1}^{\left(m\right)},z_{2,2}^{\left(m\right)},\ldots ,z_{2,K}^{\left(m\right)}\right)$, and the distances $d(z_{2,k}^{\left(m\right)},y_{2})$ are computed (with **y**_2_≡(**x**_0_,**x**_1_^*′*^,**x**_2_^*′*^)). As before, sub-trajectories are split according to their distances from the observed data, and the process is continued until trajectories reach the final time point. The resulting *K* trajectories, enriched for sub-trajectories passing close by observed data, are used as input to Equation (7) to update the parameter estimates, after which the next CE iteration begins. Figure 1 illustrates this overall process of splitting combined with the CE method. We note that setting *ρ*^*′*^=1 results in a nearly unmodified CE method as described above, and the amount of trajectory splitting can be easily tuned to the desired level by changing *ρ*^*′*^ accordingly.

#### Parameter perturbation

Both the CE method and its MS modification rely on the system’s intrinsic variability to refine parameter estimates. If a system exhibits a low level of variability, each selected subset of ⌉*ρK*⌉ trajectories will not lie much closer to the data than the other trajectories. This will result in a slowly progressing algorithm. To overcome this potential problem, we have introduced a parameter *λ*∈[0,1] which we use to independently perturb the components of the current parameter estimate for each simulated trajectory over each of the observed time intervals. We generate ${\tilde{\theta }}_{j,i,k}^{\left(m\right)}$ (*j*=1,…,*M*; *i*=1,…,*d*; *k*=1,…,*K*) as follows:

(8) $${\tilde{\theta }}_{j,i,k}^{\left(m\right)}∼U((1-\lambda ){\hat{\theta }}_{j}^{\left(m\right)},(1+\lambda ){\hat{\theta }}_{j}^{\left(m\right)}),$$

where *U*(*ab*) is a uniformly distributed random variable with minimum and maximum values *a* and *b*, respectively. We simulate each of the *d* observed time intervals for each of the *K* trajectories using independently perturbed parameters; thus, Equation (8) is evaluated *M*×*d*×*K*times for each iteration *m* of our modified CE method. Depending on the magnitude of *λ*, this procedure generates substantially more variability in each ensemble of sub-trajectories, leading to faster progression of the CE method. Although parameter perturbation is not generally used in rare event simulation, we note that a similar approach is present in iterated filtering versions of sequential Monte Carlo algorithms [27] where the perturbations allow the algorithm to escape from local minima of an objective function. In all examples presented below, we choose *λ*=.25.

### Computing MLE uncertainty estimates

An advantage of using MCEM to identify MLEs is the simplicity with which uncertainty estimates can be computed. In general, MLEs exhibit asymptotic normality; consequently, their covariance matrix can also be estimated using Monte Carlo simulation [23,28]. In order to insure that parameter confidence bounds derived from the MLE covariance matrix are positive, we introduce the transformed parameters $\omega_{j}=\log\theta_{j}(j=1,\ldots ,M)$. Due to the functional invariance property of maximum likelihood estimators, ${\hat{\omega }}_{j}=\log{\hat{\theta }}_{j}$, and by modeling $\hat{\theta }$ as a log-normally distributed random variable (which is only defined for strictly positive real numbers), $\hat{\omega }$ becomes multivariate normal with mean vector $\left(\log\theta_{1},\ldots ,\log\theta_{M}\right)$ and covariance matrix Σ. We can estimate this covariance matrix using the following expression (see [23,28] for details):

(9) $$\begin{array}{ll} -{\left(\hat{\Sigma }\right)}^{-1} & =\frac{1}{K^{\prime }}\overset{K^{\prime }}{\underset{k^{\prime }=1}{\sum }}\left\{\frac{\partial^{2}}{\partial \omega^{2}}\log f_{\omega }(x_{0},z_{k^{\prime }})\right\}\\ & \;+\frac{1}{K^{\prime }}\overset{K^{\prime }}{\underset{k^{\prime }=1}{\sum }}\left(\frac{\partial }{\partial \omega }\log f_{\omega }(x_{0},z_{k^{\prime }})\right)\\ & \;\times {\left(\frac{\partial }{\partial \omega }\log f_{\omega }(x_{0},z_{k^{\prime }})\right)}^{T}\\ & \;-\left(\frac{1}{K^{\prime }}\overset{K^{\prime }}{\underset{k^{\prime }=1}{\sum }}\frac{\partial }{\partial \omega }\log f_{\omega }(x_{0},z_{k^{\prime }})\right)\\ & \;\times {\left(\frac{1}{K^{\prime }}\overset{K^{\prime }}{\underset{k^{\prime }=1}{\sum }}\frac{\partial }{\partial \omega }\log f_{\omega }(x_{0},z_{k^{\prime }})\right)}^{T}, \end{array}$$

where {·} delimits a matrix, **a**^T^ represents the transpose of vector **a**, *f*_***ω***_(·) is equivalent to Equation (1) with exp(ω) substituted for ***θ***, $z_{k^{\prime }}$ is a reaction trajectory simulated using $\hat{\theta }=\exp(\hat{\omega })$, and *k*^*′*^ indexes only the *K*^*′*^ simulated trajectories that are consistent with the observed data. After some simplification, we arrive at:

(10) $$\begin{array}{ll} -{\left(\hat{\Sigma }\right)}^{-1} & ={\left\{-\frac{1}{K^{\prime }}\overset{K^{\prime }}{\underset{k^{\prime }=1}{\sum }}\overset{r_{k^{\prime }}}{\underset{i=1}{\sum }}\exp({\hat{\omega }}_{j})h_{j}(x_{i-1,k^{\prime }})\tau_{ik^{\prime }}\right\}}_{j}\\ & \;+\frac{1}{K^{\prime }}\overset{K^{\prime }}{\underset{k^{\prime }=1}{\sum }}{\left(r_{jk^{\prime }}-\overset{r_{k^{\prime }}}{\underset{i=1}{\sum }}\exp({\hat{\omega }}_{j})h_{j}(x_{i-1,k^{\prime }})\tau_{ik^{\prime }}\right)}_{j}\\ & \;\times {\left(r_{jk^{\prime }}-\overset{r_{k^{\prime }}}{\underset{i=1}{\sum }}\exp({\hat{\omega }}_{j})h_{j}(x_{i-1,k^{\prime }})\tau_{ik^{\prime }}\right)}_{j}^{T}\\ & \;-{\left(\frac{1}{K^{\prime }}\overset{K^{\prime }}{\underset{k^{\prime }=1}{\sum }}\left[r_{jk^{\prime }}-\overset{r_{k^{\prime }}}{\underset{i=1}{\sum }}\exp({\hat{\omega }}_{j})h_{j}(x_{i-1,k^{\prime }})\tau_{ik^{\prime }}\right]\right)}_{j}\\ & \;\times {\left(\frac{1}{K^{\prime }}\overset{K^{\prime }}{\underset{k^{\prime }=1}{\sum }}\left[r_{jk^{\prime }}-\overset{r_{k^{\prime }}}{\underset{i=1}{\sum }}\exp({\hat{\omega }}_{j})h_{j}(x_{i-1,k^{\prime }})\tau_{ik^{\prime }}\right]\right)}_{j}^{T} \end{array}$$

where {·}_*j*_ is a diagonal matrix with *j* ranging from 1 to *M* along the diagonal and (·)_*j*_ is a column vector with *j* ranging from 1 at the top-most element to *M* at the bottom. All trajectories in Equation (10) are simulated using parameter values $\hat{\theta }=\exp(\hat{\omega })$.

Upon solving Equation (10) for $\hat{\Sigma }$, we can compute the coordinates of confidence intervals and ellipses (end points and boundaries, respectively) for ***ω*** using the properties of the multivariate normal distribution. We then transform these coordinates by exponentiation to yield (strictly positive) confidence bounds for ***θ***. We note that all of the components of Equation (10) were previously required for computing MLEs using MCEM. In practice, after identifying $\hat{\theta }$, we simulate one additional ensemble of trajectories to estimate parameter uncertainties. For all examples described below, we use *K*^*′*^=10^4 ^ in this final computation.

To summarize, our proposed method for accelerating MLE identification in stochastic biochemical systems works in three steps: first, it identifies an initial parameter estimate ${\hat{\theta }}^{\mathrm{CE}}$ using a modified cross-entropy method with multilevel splitting and parameter perturbation; second, it uses this initial estimate as input to ascent-based MCEM, which is run until convergence to yield $\hat{\theta }$; third, it uses this MLE to compute parameter uncertainty estimates via Equation (10). We provide pseudo-code for the complete method below (see Algorithms 1-3), which we refer to as MCEM^2^: Monte Carlo Expectation-Maximization with Modified Cross-Entropy Method.

## Results

We now illustrate the utility of MCEM^2^ for estimating unknown parameters by applying it to data from five stochastic biochemical model systems: a pure-birth process, a birth-death process, a decay-dimerization, an auto-regulatory gene network, and a model of yeast-polarization. For each model, we first simulate a single system trajectory (with known parameters) using the SSA for a given final time *T*. Next, we extract data from this trajectory for all species at *d* equally-spaced time points, where *d*=*T*/*Δt* for a time step *Δt*. Finally, we run MCEM^2^ on the dataset and a version of the model where all information about model parameters has been withheld. Unless otherwise noted, we set the initial parameter vector for each system ${\hat{\theta }}^{\left(0\right)}$ equal to a vector of all ones. We display point estimates and confidence bounds for each simulation.

### Pure-birth process

A system for which MLEs can be computed analytically from discretely observed data is the pure-birth process, also known as a homogeneous Poisson process. The model is given by the single reaction

$$\emptyset \overset{\theta }{\to }S$$

with initial conditions **x**_0_=0. The MLE for a given dataset from this model can be easily computed by dividing the number of molecules of *S* present at the final time point by the corresponding time: $\hat{\theta }=x_{d}^{\prime }/T$. By design, both MCEM^2^ and standard ascent-based MCEM will also return this MLE (albeit at a greater computational expense), as any version of EM applied to this model ultimately reduces to the exact computation **x**_*d*_^*′*^/*T*.

**Figure 2:**
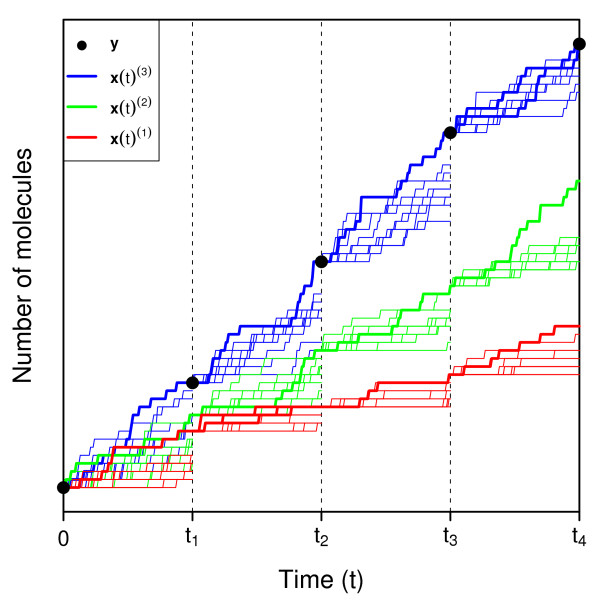
**Computing time of MCEM versus MCEM**^**2**^**for pure-birth process.** Red circles and curve fit depict computing time required for MCEM^2^ to return MLEs for the pure-birth model with ${\hat{\theta }}^{\left(0\right)}=1$ and varying *θ*^∗ ^ values. Blue circles and curve fit depict identical quantities for ascent-based MCEM. Performance of MCEM^2^ is robust to the discrepancy between initial and true parameter values, while ascent-based MCEM quickly becomes computationally intractable as the discrepancy increases.

Thus, the only potential difference between MCEM^2^ and MCEM for this system is the required computing time. To quantify this difference, we generated data for 100 pure-birth models, with *θ*^∗^, the true value, ranging from .01 to 10. For each model, we used *T*=1000 and *d*=30, giving $\mathrm{\Delta t}=33\frac{1}{3}$. We then applied ascent-based MCEM and MCEM^2^, both with ${\hat{\theta }}^{\left(0\right)}=1$, to each dataset and ran until convergence. Figure 2 displays the computing time for both methods as a function of *θ*^∗^. We see that the time required for MCEM increases dramatically as values of *θ*^∗^ depart from ${\hat{\theta }}^{\left(0\right)}$. The rapidly accelerating computational cost for MCEM is due to the rapidly decreasing likelihood of simulating a consistent trajectory as the discrepancy between ${\hat{\theta }}^{\left(0\right)}$ and *θ*^∗ ^ increases. As shown in Figure 2, MCEM is only feasible to use when ${\hat{\theta }}^{\left(0\right)}$ is within a factor of two from *θ*^∗^. In contrast, the computing time for MCEM^2^ stays approximately constant for values of *θ*^∗^ less than 1 and increases relatively slowly for values greater than 1. This cost increase is due to the simulation cost of firing more birth reactions required for larger *θ*^∗^. MCEM^2^ does not appear to suffer from a cost associated with the discrepancy between ${\hat{\theta }}^{\left(0\right)}$ and *θ*^∗^.

**Figure 3:**
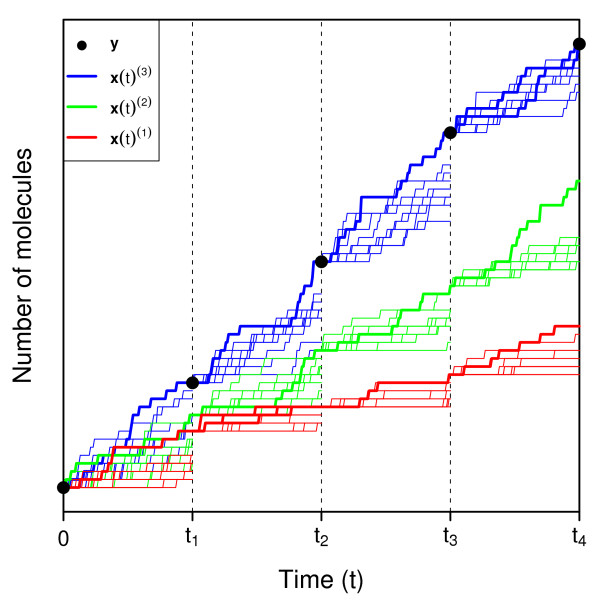
**Pure-birth process MCEM**^**2**^**MLEs and confidence intervals.** Colored circles depict MCEM^2^ MLEs normalized by true parameter values for the pure-birth model with ${\hat{\theta }}^{\left(0\right)}=1$ and varying *θ*^∗^. Error bars denote 95% confidence intervals (CIs) for each model. Out of 100 models tested, only eight (centered at blue circles) do not overlap the true parameter values (green line) whereas the remaining 92 (centered at red circles) enclose the truth. This agrees well with the expected 95/100.

We next investigated the accuracy of MCEM^2^ uncertainty estimates. Figure 3 shows the normalized MCEM^2^ MLEs with 95% confidence intervals (CIs) for all models. Out of 100 CIs, only eight (denoted by blue circles) do not overlap the true values. This figure matches well with the expected number of missed overlaps (100×(1−.95)=5) and suggests that our asymptotic normality assumption for deriving MLE confidence bounds is valid. We note that the relative magnitudes of the CIs decrease with increasing *θ*^∗^; this is due to the diminishing effect of noise on the system as the average number of reaction firings per unit time increases.

### Birth-death process

The second model doubles the number of reactions of the pure-birth process by adding a degradation reaction. The birth-death process takes the form:

$$\begin{array}{cc} \emptyset & \overset{\theta_{1}}{\to }S\\ S & \overset{\theta_{2}}{\to }\emptyset \text{.} \end{array}$$

**Figure 4:**
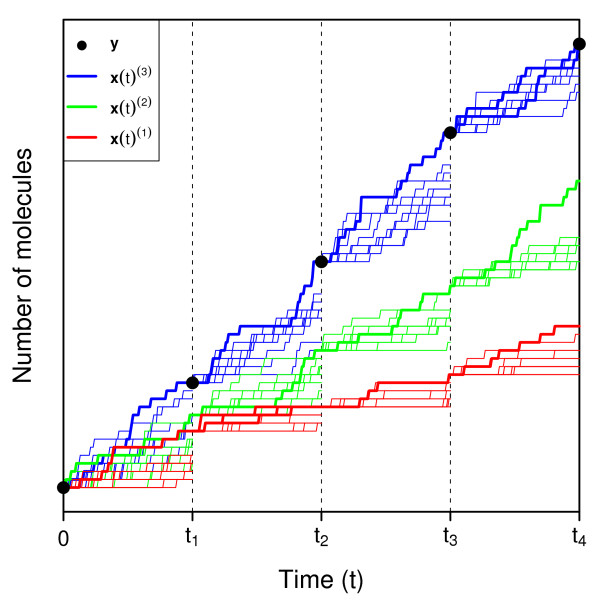
**Birth-death process MCEM**^**2**^**parameter estimate progression.** Green and blue bold lines denote MCEM^2^ parameter estimates ${\hat{\theta }}_{1}$ and ${\hat{\theta }}_{2}$, respectively, as a function of iteration number. True parameter values are marked by green and blue horizontal dotted lines. The cross-entropy phase completes in three iterations (gray shaded region), followed by 234 iterations of MCEM until convergence (black vertical line). An additional 100 iterations of MCEM are included to illustrate the diminishing returns from running the algorithm beyond convergence. Although the parameter estimates from the first MCEM iteration are far from the true values, their ratio is nearly correct and this ratio is preserved as the estimates are refined toward the true values.

The presence of a single first order reaction (degradation) renders the analytical calculation of MLEs infeasible. Furthermore, computational parameter identification for the birth-death process is significantly more challenging than for the pure-birth process. This challenge stems from the degeneracy present in a discretely observed dataset: the net increase of a single molecule of *S* can result from any combination of *r* + 1*R*_1_ and *rR*_2_ reaction firings (where *r* is a non-negative integer). To evaluate MCEM^2^ on this system, we first generated single trajectory data for a model with ***θ***^∗^=(1,.06) and **x**_0_=17, where the system starts in stochastic equilibrium. We used *T*=200 and *d*=40, giving *Δt*=5. Figure 4 displays the progression of ${\hat{\theta }}_{1}$ and ${\hat{\theta }}_{2}$ as a function of MCEM^2^ iteration. The modified cross-entropy phase of the algorithm required only three iterations (labeled -2,-1,0), transforming ${\hat{\theta }}^{\left(0\right)}=(1,1)$ to ${\hat{\theta }}^{\left(3\right)}=(4.24,.28)$. From this point onward, the subset of trajectories given by *ρ*=.001 were consistent with the data, and the MCEM phase of the algorithm further modified the parameters to their final values $\hat{\theta }=(1.446,\text{.}093)$, which were reached upon satisfying the convergence criterion (marked by black vertical line). Figure 4 also includes the results from an additional 100 iterations of MCEM to illustrate the diminishing returns from running the algorithm beyond the convergence criterion. Throughout the MCEM phase, we note that the ratio ${\hat{\theta }}_{2}^{\left(n\right)}/{\hat{\theta }}_{1}^{\left(n\right)}\approx .065$, indicating that multiple parameter values satisfying this ratio are sufficient to generate consistent trajectories. Nevertheless, Figure 4 demonstrates that substantial parameter refinement is achieved by running MCEM to convergence.

**Figure 5:**
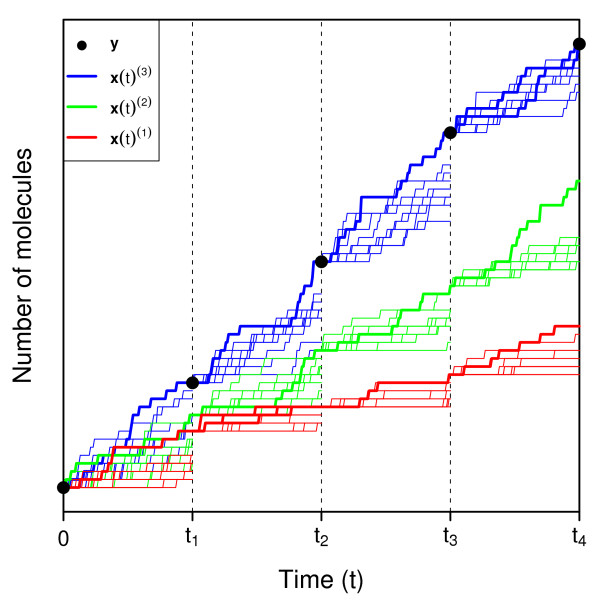
**Effects of birth-death dataset size on parameter estimates and MCEM**^**2**^**uncertainty.** Each panel displays MCEM^2^ and SGD birth-death MLEs (red and blue circles, respectively) as well as Poisson method point estimates (orange circles) versus the true parameter values (green circles), along with MCEM^2^ 68%, 95%, and 99% confidence ellipses (black curves ranging in size from smallest to largest, respectively). **A**, **B**, **C**, and **D** display results for datasets of 40, 60, 80, and 100 data points, respectively. The three methods tested identified parameters with comparable accuracy across all datasets. As the numbers of data points increase, the MCEM^2^ MLEs get closer to the truth and the confidence ellipses shrink in size. The green sloped line plots the ratio $\theta_{2}^{*}/\theta_{1}^{*}$, highlighting that the uncertainties of the parameter ratio are lower than the uncertainties of the parameter magnitudes. For all datasets, the 95% confidence ellipse encloses the true parameter values.

Next, we investigated the effect of appending data at additional time points to the original data set. Figure 5 illustrates results from the original and three expanded datasets, all with *Δt*=5. We display the MCEM^2^ MLEs along with 68%, 95%, and 99% confidence ellipses (warped due to exponentiation—see Methods) that represent parameter uncertainty as a function of both parameters. We see that as *d* increases, $\hat{\theta }$ approaches ***θ***^∗^ until at *d*=100 they are approximately equal. This trend demonstrates the increasing accuracy of MLEs with increasing *d*. Furthermore, although the true parameter values are always contained within the 95% confidence ellipses, all of the ellipses shrink in size as *d* increases. This behavior indicates the reduction in estimate uncertainty resulting from the addition of data points. Finally, all of the ellipses are clearly skewed, with major axes nearly overlapping the line passing through the origin whose slope is the ratio of the true parameter values (.06/1). This geometry shows that most of the uncertainty involves the magnitude of the parameters, whereas their ratio can be determined confidently from relatively few data points. We note that the computational run time of MCEM^2^ (1 × Intel 3 GHz processor) on each of the four datasets was approximately the same: one hour.

We also compared MCEM^2^ performance to that of two recent methods: an MLE method utilizing reversible jump Markov chain Monte Carlo coupled with stochastic gradient descent (“SGD”) [4] and a Bayesian method using a Poisson process approximation (“Poisson”) [14]. For the former, we used the provided MATLAB package to run SGD with the maximum number of iterations set to 500 and the initial sample size set to 600 (incrementing by 500 every 10 iterations). For the latter, we used the provided C code from the author’s web site implementing the program to run the Poisson method with tuning parameter .05 and total number of iterations 10^7^ (with 10^5^ burn-in iterations and 10^4^ thinning interval). These options were chosen to yield sufficient mixing and convergence properties as evidenced by the diagnostic plots from the R package. We then computed the mean value of each parameter to arrive at point estimates. As with MCEM^2^, we set ${\hat{\theta }}^{\left(0\right)}=(1,1)$ for both methods. Figure 5 displays the SGD and Poisson method results for the four birth-death process datasets. When compared to MCEM^2^, all three methods identified parameters with comparable accuracy, with SGD and Poisson methods performing better when *d*=40 and *d*=60 and MCEM^2^ performing better when *d*=80 and *d*=100. The confidence ellipses generated by the Poisson method were very similar in appearance to those of MCEM^2^, conveying the same information regarding the ratios of the two parameters (not shown). As noted above, the SGD method did not provide parameter uncertainty estimates. Regarding run time, the Poisson method required between 20 and 60 minutes to identify parameters for the four datasets, while the SGD method needed between 30 minutes and several days (the latter time due to a lack of convergence when using the *d*=100 dataset).

**Figure 6:**
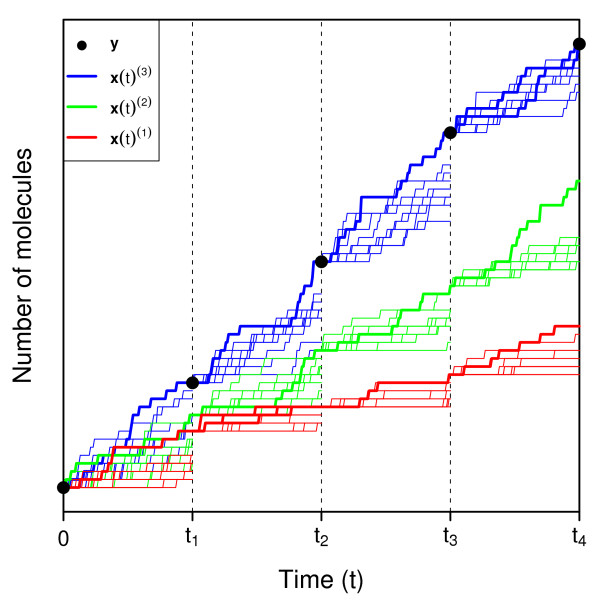
**Effects of decreasing birth-death equilibrium on MCEM**^**2**^**and Poisson method performance.** Boxplots (displaying median, first and third quartiles, and most extreme data point within 1.5 × the interquartile range from the box) summarize mean relative errors of MCEM^2^ and the Poisson method applied to 20 birth-death datasets for each of five models (true parameter values listed on x-axis). Models are sorted in decreasing order of the equilibrium value of *S*, ranging from 5 to .1. MCEM^2^ performance does not vary appreciably across the different models, while the Poisson method exhibits increasing error with decreasing equilibrium value.

We next modified the birth-death process such that the equilibrium value of species *S* gradually approached zero. Specifically, we created five models with true parameter values $\theta_{1}^{*}=.5$ and $\theta_{2}^{*}$ taking the increasing values (.1, .5,1, 2.5, 5). To insure that each system started roughly at stochastic equilibrium, we also set **x**_0_ to each of the following values (listed in order): (5,1,1,1,1). We then generated 20 independent datasets for each of the five models, using *T*=25 and *d*=25. Figure 6 displays boxplots of the mean relative error (calculated as in [4]: $\frac{1}{M}\overset{M}{\underset{j=1}{\sum }}|{\hat{\theta }}_{j}-\theta_{j}^{*}|/\theta_{j}^{*}$) when applying MCEM^2^ and the Poisson method to each of these datasets. Although both methods perform equally well for the first three models (when the equilibrium value of *S*≥.5), MCEM^2^ clearly identifies parameters more accurately than the Poisson method for the last two datasets (when the equilibrium values of *S* are .2 and .1, respectively). This result illustrates the gradual loss of accuracy of the Poisson approximation for systems in which a species tends stochastically to zero. In contrast, MCEM^2^, which generates exact system trajectories using the SSA, experiences no such loss of accuracy. Unfortunately, we were unable to evaluate SGD on these modified birth-death process datasets, as the MATLAB package consistently terminated with an error related to the zero molecule count of S.

### Decay-dimerization model

**Figure 7:**
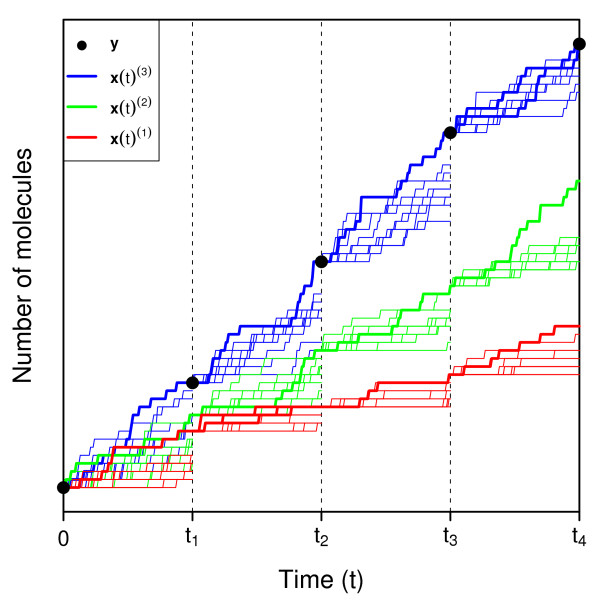
**Effects of decay-dimerization model structure on MCEM**^**2**^**, Poisson method, and SGD performance.** Boxplots summarize mean relative errors of the three methods applied to 10 decay-dimerization datasets for each of two three-reaction models. The two models differ only in their third reaction (listed on x-axis); the first model contains a reversible dimerization, while the second model does not. MCEM^2^ and the Poisson method perform similarly across both models, while SGD consistently incurs an infinite mean relative error (due to the estimate of *θ*_1 _ quickly tending to infinity) when applied to the second (irreversible) model.

The next system contains reactions involving species decay and dimerization. We begin with the following three reactions, where the dimerization step is reversible:

$$\begin{array}{ll} & S_{1}\overset{\theta_{1}}{\to }\emptyset \\ S_{1}+ & S_{1}\overset{\theta_{2}}{\to }S_{2}\\ & S_{2}\overset{\theta_{3}}{\to }S_{1}+S_{1} \end{array}$$

with **x**_0_=(40,0). We generated ten single-trajectory datasets for a model where ***θ***^∗^=(.2, .04, .5), using *T*=5 and *d*=25. We then modified the model such that the dimerization step is no longer reversible, leading to the following description:

$$\begin{array}{ll} & S_{1}\overset{\theta_{1}}{\to }\emptyset \\ S_{1}+ & S_{1}\overset{\theta_{2}}{\to }S_{2}\\ & S_{2}\overset{\theta_{3}}{\to }\emptyset \end{array}$$

with all other properties unchanged. We again generated ten single-trajectory datasets for this model. Finally, we evaluated MCEM^2^, the Poisson approximation method, and SGD on each of the 20 datasets. Figure 7 displays the results for each of the methods in terms of mean relative error. We see that MCEM^2^ and the Poisson method perform very similarly in terms of accuracy (as well as run time: between 3 and 10 minutes for both models), with a slightly higher error for the irreversible model. In contrast, use of SGD results in higher errors for both models, with the irreversible model consistently yielding estimates with infinite error. This latter error is due to the estimate of *θ*_1_ quickly tending to infinity, regardless of how small we set the initial gradient descent step size. These results highlight a significant limitation of the SGD method: in order to generate a diversity of consistent trajectories, there must exist combinations of reactions that do not alter species counts. The reversible decay-dimerization model contains such a combination (reactions 2 and 3), while the irreversible model does not, leading to a divergent gradient descent.

**Figure 8:**
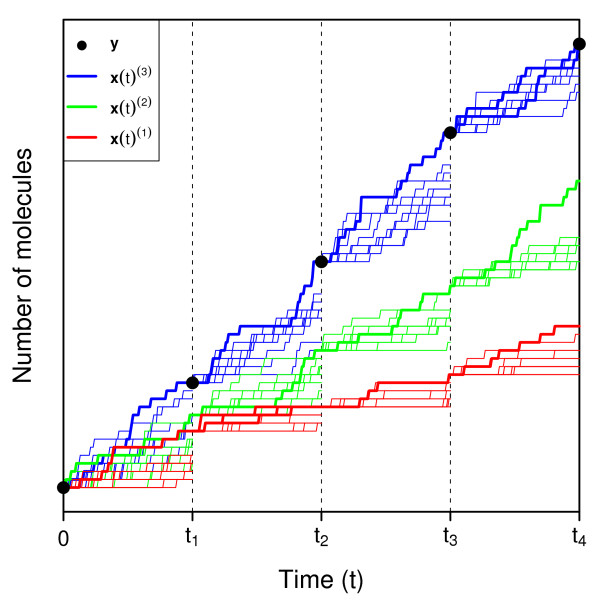
**Decay-dimerization dataset.** Red, green, and blue circles depict initial system states and five data points for species *S*_1_, *S*_2_, and *S*_3_, respectively. This dataset is sparsely observed, as species *S*_1 _ changes substantially between *t*=0 and the first observed time point.

To further explore the ability of MCEM^2^ to estimate parameters for a decay-dimerization, we introduced a third model which adds a conversion reaction to the reversible model above. Previously analyzed in [29], the precise system description is as follows:

$$\begin{array}{ll} & S_{1}\overset{\theta_{1}}{\to }\emptyset \\ S_{1}+ & S_{1}\overset{\theta_{2}}{\to }S_{2}\\ & S_{2}\overset{\theta_{3}}{\to }S_{1}+S_{1}\\ & S_{2}\overset{\theta_{4}}{\to }S_{3} \end{array}$$

with **x**_0_=(1000,10,10). We generated single trajectory data for a model where ^***θ***∗^=(.2, .04, .5, 1), using *T*=.1 and *d*=5. Figure 8 shows the data points for each of the three species. Given that *Δt*=.02, hundreds of reactions occur before the first observed time point. As the system evolves closer to its steady state, the number of reaction firings decreases, with only dozens of reactions firing between the last two time points. We note that the initial propensity for reaction *R*_2 _ is nearly 4000 times larger than the propensity of its backwards counterpart *R*_3_; consequently, we expect observed data to reflect relatively few *R*_3_ firings (and thus contain relatively little information about $\theta_{3}^{*}$).

**Figure 9:**
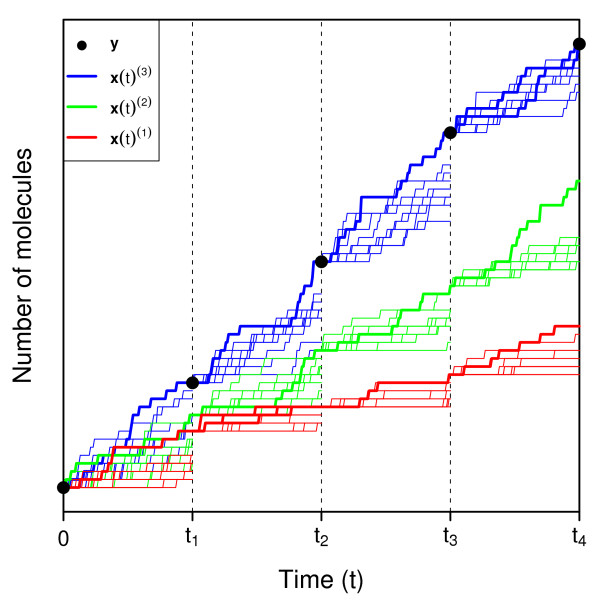
**Effects of parameter perturbation on decay-dimerization cross-entropy phase.** Red, blue, green, and orange lines represent MCEM^2^ parameter estimates ${\hat{\theta }}_{1}$, ${\hat{\theta }}_{2}$, ${\hat{\theta }}_{3}$, and ${\hat{\theta }}_{4}$, respectively, as a function of cross-entropy (CE) phase iteration number. Solid lines display parameter values observed using perturbation (*λ*=.25), while dotted lines depict parameter values obtained without perturbation (*λ*=0). Perturbation substantially accelerated completion of the CE phase, both in number of iterations (23 versus 152) and, more strikingly, in simulation time (59 s versus 32 min). Final CE phase parameter estimates were approximately the same whether or not perturbation was used.

To investigate the impact of parameter perturbation on the performance of MCEM^2^, we estimated parameters from this decay-dimerization dataset using both *λ*=.25 (default) and *λ*=0 (no perturbation). Figure 9 shows the progression of each parameter during the cross-entropy phase of the algorithm for both default perturbation (solid line) and no perturbation (dotted line). With *λ*=.25, the CE phase required only 23 iterations before beginning MCEM, whereas setting *λ*=0 increased the number of CE iterations to 152. More importantly, the CE phase computing times for perturbation and no perturbation were 59 s and 32 min, respectively, resulting in a ∼33-fold speedup when perturbing parameters. The reason for this large reduction in computational time is due to the larger parameter values explored by the CE phase without perturbation (see ${\hat{\theta }}_{1}$ and ${\hat{\theta }}_{3}$), which equates to simulating trajectories with many more reaction firings. By using perturbation, MCEM^2^ appears to navigate the parameter space more efficiently and hence require much less computational time. We note that three of the four parameters reach approximately the same values at the end of the CE phase in the perturbed and non-perturbed cases, with ${\hat{\theta }}_{3}$ providing a slight exception. However, as we show below, the large uncertainty associated with ${\hat{\theta }}_{3}$ prevents us from determining whether this parameter is substantially different between the two cases. We thus conclude that perturbation does not systematically alter the final parameter estimates returned by the CE phase.

**Figure 10:**
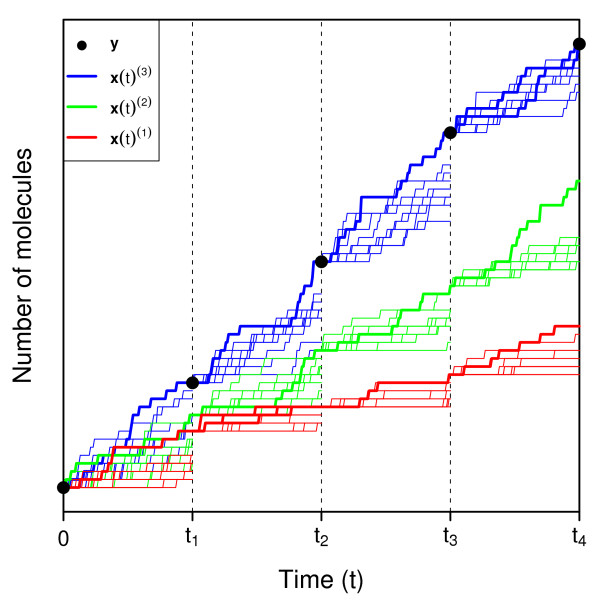
**Parameter estimation results for decay-dimerization model.** Each panel displays MCEM^2^ MLEs (red circles) versus the true parameter values ***θ***^∗^=(.2, .04, .5, 1) (green circles), along with 68%, 95%, and 99% confidence ellipses. All six pairwise parameter comparisons are shown. The mean relative error for MCEM^2^ was 22.8%. All MCEM^2^ confidence ellipses enclose the true parameter values, and uncertainty is relatively low for all estimates except ${\hat{\theta }}_{3}$.

Figure 10 displays the MLEs and pairwise confidence ellipses computed by MCEM^2^ when applied to this decay-dimerization dataset. Specifically, MCEM^2^ returned $\hat{\theta }=(.220,\text{.}039,\text{.}110\text{,}1\text{.}006)$, which represents a 22.8% mean relative error when compared to the truth. For all combinations of parameters, the corresponding 68% confidence ellipses enclose the true parameter values, and apart from ${\hat{\theta }}_{3}$ these ellipses are relatively compact. As noted above, the uncertainty associated with reaction *R*_3_ is much larger than for the other reactions, confirming our hypothesis that the dataset contains substantially less information about the backwards rate of the dimerization.

### Auto-regulatory gene network

To further compare MCEM^2^ to the Poisson method and SGD, we tested all methods on a system for which SGD was previously shown to perform well: a prokaryotic auto-regulatory gene network [4]. This system contains the following eight reactions, organized as four reversible pairs:

$$\begin{array}{l} \;\mathrm{DNA}+P_{2}\overset{\theta_{1}}{\to }\text{DNA}\text{-}P_{2}\\ \;\text{DNA}\text{-}P_{2}\overset{\theta_{2}}{\to }\text{DNA}+P_{2}\\ \;\text{DNA}\overset{\theta_{3}}{\to }\text{DNA}+\text{mRNA}\\ \;\text{mRNA}\overset{\theta_{4}}{\to }\emptyset \\ \;P+P\overset{\theta_{5}}{\to }P_{2}\\ \;P_{2}\overset{\theta_{6}}{\to }P+P\\ \;\mathrm{mRNA}\overset{\theta_{7}}{\to }\text{mRNA}+P\\ \;P\overset{\theta_{8}}{\to }\emptyset \text{,} \end{array}$$

where *DNAPP*_2_, and *mRNA * represent DNA promoters, protein gene products, protein dimers, and messenger RNA molecules, respectively. We set **x**_0_≡(*DNADNAP*_2_*mRNAPP*_2_) = (7, 3, 10, 10, 10) and generated single trajectory data using ***θ***^∗^=(.1, .7, .35, .3, .1, .9, .2, .1) with *T*=50 and *d*=100. Using the same options as before, we applied MCEM^2^ and SGD to this dataset using ${\hat{\theta }}^{\left(0\right)}=(1,1,1,1,1,1,1,1)$. We also applied the Poisson method using total number of iterations 10^8^, with 10^6^ burn-in iterations and 10^5^ thinning interval (these values were increased from before to preserve adequate mixing and convergence). As in previous examples, we initially used *ρ*=.001 in the CE phase of MCEM^2^. However, this proportion was not small enough to enable the generation of ⌉*ρK*⌉ consistent trajectories for this system (and thus to progress to MCEM). To compensate, we re-ran MCEM^2^ using *ρ*=.0001 and *K*=10^5^. This time, the CE phase completed easily in five iterations.

**Figure 11:**
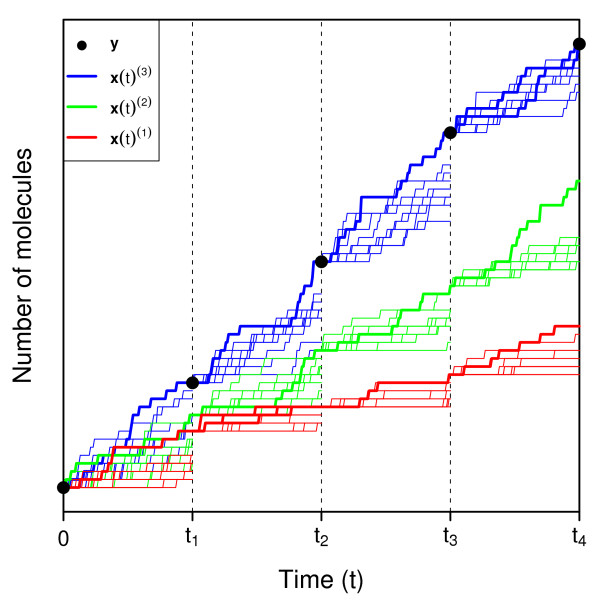
**Parameter estimation results for auto-regulatory gene network.** Each panel displays MCEM^2^ and SGD MLEs and Poisson method point estimates computed using ${\hat{\theta }}^{\left(0\right)}=(1,1,1,1,1,1,1,1)$ (red, blue, and orange circles, respectively), true parameter values (green circles), and MCEM^2^ 68%, 95%, and 99% confidence ellipses. **A**, **B**, **C**, and **D** compare the four reversible pairs of reactions in the system. Mean relative errors for MCEM^2^, SGD, and the Poisson method were 52%, 20%, and 30%, respectively. MCEM^2^ 95% confidence ellipses enclosed all true parameter values except $\theta_{5}^{*}$ and $\theta_{6}^{*}$; like the birth-death system, their skew indicates that the uncertainties of the parameter ratios are lower than the uncertainties of the parameter magnitudes.

Figure 11 displays MLEs for all three methods, as well as the MCEM^2^ pairwise confidence ellipses for the four reversible reaction pairs. We see that all methods estimate most parameters with approximately equal accuracy, although MCEM^2^ and SGD more accurately determine $\theta_{1}^{*}$ and $\theta_{2}^{*}$, while the Poisson method and SGD more accurately determine $\theta_{5}^{*}$ and $\theta_{6}^{*}$. The mean relative errors for MCEM^2^, SGD, and the Poisson method were 52%, 20%, and 30%, respectively. The MCEM^2^ 95% confidence ellipses enclose all true parameters except $\theta_{5}^{*}$ and $\theta_{6}^{*}$, and as in the birth-death system, all ellipses attribute most of the uncertainty to knowledge of the magnitudes of parameter pairs rather than their ratios. The ellipses generated by the Poisson method were skewed in the same manner, conveying similar information regarding parameter ratios (not shown). Regarding run times, the Poisson method was by far the fastest, requiring only 1.5 hours to estimate parameters. In contrast, SGD and MCEM^2^ required 2.3 and 8.7 days on a single processor, respectively, to complete.

In [4], the SGD method was also used to identify parameters from datasets where only a subset of species were observed. We modified our original dataset by removing observed molecule counts for species *DNA* and *DNA*−*P*_2_ at all time points except *t*=0 and re-ran MCEM^2^. Upon convergence, we obtained $\hat{\theta }=(0.043,0.538,\;0.302,\;0.377,\;0.301,\;3.103,\;0.494,\;0.243)$ for a 107% mean relative error. This roughly translates to a 2-fold increase in relative error due to a 40% decrease in observed data points. Unfortunately, we were not able to compare to the performances of SGD or the Poisson method, as neither implementation was executable on datasets with missing species.

### Yeast-polarization model

**Figure 12:**
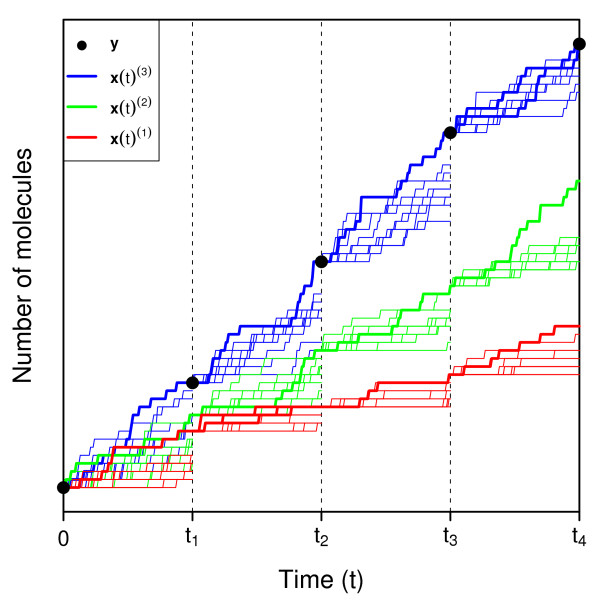
**Yeast-polarization dataset.** Colored circles depict initial system states and 15 data points for all seven species. Like the decay-dimerization dataset, these data are sparsely observed, particularly with respect to species *G*, *G*_*a*_, and *G*_*bg*_ between *t*=0 and the first observed time point.

The final system we used to evaluate MCEM^2^ models the pheromone-induced G-protein cycle in *Saccharomyces cerevisiae*[18,30]. This model consists of the following eight reactions:

$$\begin{array}{ll} & \emptyset \overset{\theta_{1}}{\to }R\\ & R\overset{\theta_{2}}{\to }\emptyset \\ L+ & R\overset{\theta_{3}}{\to }\mathrm{RL}+L\\ & \mathrm{RL}\overset{\theta_{4}}{\to }R\\ \mathrm{RL}+ & G\overset{\theta_{5}}{\to }G_{a}+G_{\mathrm{bg}}\\ & G_{a}\overset{\theta_{6}}{\to }G_{d}\\ G_{d}+ & G_{\mathrm{bg}}\overset{\theta_{7}}{\to }G\\ & \emptyset \overset{\theta_{8}}{\to }\mathrm{RL}\text{,} \end{array}$$

where *RL*, and *RL* represent pheromone receptors, ligands, and receptor-ligand complexes, respectively. Species *G* corresponds to a G-protein with separate subunits *G*_*a*_*G*_*bg*_, and *G*_*d*_. We used **x**_0_≡(*RLRLGG*_*a*_*G*_*bg*_*G*_*d*_)=(500,4,110,300,2,20,90) and generated single trajectory data for ^***θ***∗^=(.38, .04,.082, .12, .021, .1, .005, 13.21) using *T*=5 and *d*=15. Figure 12 displays the data points for all species. As with the final decay-dimerization model, this dataset is sparsely observed, particularly with respect to species *GG*_*a*_, and *G*_*bg*_ at early time points.

We first tested MCEM^2^ on this dataset with ${\hat{\theta }}^{\left(0\right)}=$(1,1,1,1,1,1,1,1) and *ρ*=.001. As with the auto-regulatory gene network, this value of *ρ* was not small enough to enable the generation of ⌉*ρK*⌉ consistent trajectories. Given the greater computational expense of simulating the yeast-polarization model, we decided against reducing *ρ*and increasing *K* further until the CE phase converged. Instead, we prematurely terminated the CE phase once the distance from the observed data reached a steady minimum value, and proceeded to MCEM. This occurred at ∼70 iterations, when *δ*^(*m*)^≈.033 (see Methods). Although we expected premature entry into MCEM to increase the time required to simulate consistent trajectories in the first few iterations, we did not notice an appreciable trend and MCEM converged (defined here as when the change in conditional log-likelihood was less than .005 for at least one iteration) in 55 iterations. The resulting MLEs and available 68% confidence intervals (CIs) are displayed in Table 1. MCEM^2^ achieved a 34.7% mean relative error, and all determined CIs enclosed the corresponding true parameter values.

**Table 1:** Yeast-polarization model: parameter estimates and mean relative error (% Error) for MCEM^2^ MLEs, SGD MLEs (SGD_2_ initialized with values exhibiting 12% mean relative error), and Poisson method point estimates, along with the MCEM^2^ and Poisson method 68% confidence intervals (CIs) for each parameter

**Method**	**Type**	***θ***_1_	***θ***_*2*_	***θ***_*3*_	***θ***_*4*_	***θ***_*5*_	***θ***_*6*_	***θ***_*7*_	***θ***_*8*_	**% Error**
	True	**.38**	**.04**	**.082**	**.12**	**.021**	**.1**	**.005**	**13.21**	
MCEM^2^	Lower	n/a	.014	.076	n/a	.021	.089	.005	7.386	
	MLE	.0005	.026	.081	.0009	.022	.104	.006	11.479	34.7
	Upper	n/a	.048	.087	n/a	.024	.122	.006	17.839	
	Lower	.002	.003	.080	.0001	.018	.069	.004	.0005	
Poisson	Mean	2.233	.020	.086	.016	.019	.083	.005	1.719	93.3
	Upper	4.749	.033	.092	.027	.021	.095	.005	3.972	
SGD_1_	MLE	1.000	.798	.334	1.425	Inf	.591	.039	1.024	Inf
SGD_2_	MLE	.439	.043	.042	3.241	Inf	.029	.003	2.649	Inf

We next tested the Poisson method on the yeast-polarization dataset, using ${\hat{\theta }}^{\left(0\right)}=(1,1,1,1,1,1,1,1)$ and the same options as in the auto-regulatory gene network example. Table 1 displays the resulting parameter estimates, along with the 68% CIs. Compared to MCEM^2^, the Poisson method incurred a 2.7-fold higher mean relative error, and only half of the CIs enclosed the true parameter values. Although less accurate for this example, the Poisson method required substantially less run time than MCEM^2^: three hours versus ∼30 days on a single processor. This difference reflects the significant cost of simulating trajectories with the SSA rather than using a Poisson approximation.

Finally, we tested SGD on the yeast-polarization dataset using the same options as in previous examples (“SGD_1_”). As in the decay-dimerization model, the SGD estimate for one of the parameters (*θ*_5_) tended to infinity within nine steps of the algorithm (and thus resulted in an infinite mean relative error), even when using an initial gradient descent step size as small as 10^−6^(see Table 1). We then retested SGD using initial parameter values much closer to the truth (12% mean relative error): ${\hat{\theta }}^{\left(0\right)}=$(.461,.047, .086, .123, .015, .085, .005, 12.299) and other options unchanged (“SGD_2_”). This is in contrast to MCEM^2^ and SGD_1_, which were run with initial parameter values set to a vector of ones. As before, the same parameter estimate tended to infinity, although this time 46 steps were required to do so. Although the yeast-polarization system contains combinations of reactions that leave species numbers unchanged, they are evidently not sufficient to allow adequate trajectory generation for a non-divergent gradient descent. Table 1 displays both sets of SGD parameter estimates without CIs, as the method does not provide uncertainty estimates.

## Discussion

This work presents MCEM^2^, a novel enhancement of MCEM that accurately estimates unknown parameters of stochastic biochemical systems from observed data. MCEM^2^ combines a state of the art, adaptive implementation of MCEM (ascent-based MCEM) with algorithms from rare event simulation (the CE method and multilevel splitting) to substantially accelerate parameter estimation. Unlike a previous application of the EM algorithm to stochastic parameter estimation [13], which performs an error-prone estimation of likelihood via reaction modification, MCEM^2^ concludes by executing an unmodified MCEM iteration. This places MCEM^2^ on solid theoretical foundations, with the CE phase of the algorithm serving only to accelerate the eventual MCEM phase. We note that this acceleration is essential for the method to be useful, as the use of unmodified MCEM is computationally tractable only when initial parameter estimates are close to the true values (see Figure 2). We demonstrated that the addition of a third technique, parameter perturbation, accelerated execution of MCEM^2^ even further, without noticeable effects on the resulting parameter estimates. This was true even when using values of *λ* (denoting the maximum percent perturbation applied to each parameter) other than .25 (results not shown). If we decreased *λ*toward zero, the CE phase ran progressively slower with the same final results. If instead we increased *λ* toward one, the CE phase ran faster for some models while requiring larger sample sizes to converge (and thus running slower) for others. This latter effect is due to the increased noise conferred by using larger parameter perturbations. Ultimately, we found that by setting *λ*=.25, we achieved a useful speedup for all models tested without imposing larger sample size requirements.

MCEM^2^ requires selection of three additional user-defined quantities to achieve good performance: *d*(**zy**), an observed data distance function; *K*, the total number of simulated trajectories; and *ρ*, the proportion of trajectories selected that are closest to observed data. For the former, we chose a normalized *L*_1 _ distance, intended to provide approximately equal weight to each of the system species. Although this distance function yielded excellent performance, other functions are certainly possible (e.g. sum of squared deviations). However, we note that work performed using the related approximate Bayesian computation (ABC) methods suggests that the resulting parameter estimates are not sensitive to the choice of the distance metric [31]. The latter two parameters dictate the number of trajectories ⌉*ρK*⌉ used to refine parameter estimates at each step of the CE phase. Additionally, in order for the CE phase to converge, the proportion of simulated trajectories that are consistent with data in each time interval must be ≥*ρ*in the final step. In the first three models tested in this work, we found *K*=10^4^ and *ρ*=.001 to be sufficient for relatively fast completion of the CE phase. For the auto-regulatory gene network model, these values were not adequate to enable the generation of (100×*ρ*)% consistent trajectories, and we increased *K* to 10^5^and lowered *ρ*to .0001 to achieve convergence. Similarly, the original values were not sufficient for the yeast-polarization model, although we chose to terminate the CE phase prematurely rather than incur an additional simulation cost by increasing *K*. This practice did not noticeably impact the time required to execute MCEM iterations, which suggests that the actual proportion of simulated consistent trajectories was only slightly less than .001. In general, we suggest starting with *K*=10^4^and *ρ*=.001 and increasing *K* only if computationally favorable. Otherwise, we would recommend terminating the CE phase when the distance from the observed data reaches a steady minimum value. We note that the CE phase of MCEM^2^ with early termination resembles the ABC method of Toni *et al.*[31], with two important differences. First, the ABC method requires a user-defined threshold for selecting simulated trajectories based on their distances from observed data, whereas MCEM^2^ automatically chooses this threshold using the parameter *ρ*. Second, the method of Toni *et al.* requires accurate prior bounds on parameter values, whereas MCEM^2^ needs no prior parameter information. This latter difference also sets our method apart from the SML and histogram-based approaches for identifying MLEs [2,12], both of which require prior parameter bounds to execute a genetic algorithm.

Another important advantage of MCEM^2^ over existing MLE methods is the ease with which it can estimate parameter uncertainty. Existing MLE methods return parameter point estimates, but these estimates carry no measures of confidence or interdependency. In contrast, MCEM^2^ returns a multivariate parameter uncertainty estimate. This estimate indicates correlations between particular parameter estimates (see Figures 5 and 11), along with measures of the information content of the observed data for each unknown parameter (compare confidence ellipses of ${\hat{\theta }}_{3}$ to other parameters in Figure 10). In order to generate uncertainty estimates, MCEM^2^ assumes that MLEs are multivariate log-normally distributed, which can be shown to be true as the number of data points increases asymptotically. However, 30 data points appear to be sufficient to satisfy this assumption (Figure 3), with possibly as few as five being acceptable (decay-dimerization dataset: Figure 10). Of the pairwise confidence ellipses generated in this work (describing estimates of the birth-death process, decay-dimerization, and auto-regulatory gene network), we observed only one instance where the true parameter pair did not reside within the 99% confidence ellipse (parameters *θ*_5_ and *θ*_6_ of auto-regulatory gene network: Figure 11C). Nevertheless, we note that the true parameter values in this case line up with the major axis of the corresponding ellipse, suggesting that MCEM^2^ was still able to correctly identify the ratio of the parameters. We note that Bayesian approaches like the Poisson approximation method also generate multivariate parameter uncertainty estimates which provide similar information to that given by MCEM^2^.

We compared MCEM^2^ to the recently proposed Poisson approximation and SGD approaches by applying all three methods to four examples: birth-death process, decay-dimerization, auto-regulatory gene network, and the yeast-polarization model. Overall, the results demonstrate that MCEM^2^ performs relatively well for all examples. The first example illustrated that predictions made by the Poisson approximation method increasingly lose accuracy as species molecule counts tend to zero. MCEM^2^ avoids any such accuracy loss due to its exact simulation of consistent trajectories. The second example illustrated a limitation of the SGD method: to function properly, it requires systems to contain combinations of reactions that do not alter species counts. MCEM^2^ (as well as the Poisson method) imposes no such requirement. The divergence of the gradient descent in the yeast-polarization model also suggests that the mere presence of these combinations of reactions are not sufficient to lead to good SGD performance.

When functioning correctly on larger systems, an advantage of both SGD and the Poisson approximation method over MCEM^2^ is their lower required computational time. In particular, SGD ran 3.78-fold faster than MCEM^2^ for the auto-regulatory gene network, and the Poisson method ran an additional 36.8-fold faster than SGD. On the yeast-polarization model, the Poisson method ran 240-fold faster than MCEM^2^. These speed-ups are due to both methods’ “simulation free” approaches for generating consistent trajectories, which is advantageous for computationally expensive models. Although the CE phase of MCEM^2^ typically completes in only a few iterations, the MCEM phase can require ≥100 iterations, with each iteration modifying the parameter estimates only slightly. Thus, a modified version of MCEM that takes larger steps in parameter space would further accelerate convergence. Such modifications have previously been described in the literature [28]; consequently, current work focuses on incorporating these modifications into MCEM^2^. We note that one simple way to reduce the computational time required by MCEM^2^ is to simulate trajectories in parallel, using either clusters of CPUs (central processing units) or GPUs (graphics processing units). Since each consistent trajectory can be simulated independently of all others, the computation time of each MCEM^2^ iteration can in principle be reduced to the longest time required to simulate a single consistent trajectory.

One final enhancement that would broaden the applicability of MCEM^2^ involves accommodating measurement error in the observed data. Implementing this enhancement would be relatively straightforward given probabilistic error with known distribution. In this case, we could simply replace the indicator function in Equation 4b with the corresponding density function of the error, given a simulated trajectory. This modification would substantially improve the efficiency of MCEM^2^, as any simulated trajectory could now have a nonzero likelihood of generating the observed data (and thus all trajectories could be consistent with observed data). Future work will focus on incorporating this enhancement into MCEM^2^.

## Conclusions

In this work, we developed Monte Carlo Expectation-Maximization with Modified Cross-Entropy Method (MCEM^2^), a novel method for maximum likelihood parameter estimation of stochastic biochemical systems. Through applying MCEM^2^ to five example systems, we demonstrated its accurate performance and distinct advantages over existing methods. We expect these advantages to permit analysis of larger and more realistic biochemical models, ultimately providing an improved mechanistic understanding of important biological processes.

## Algorithm 1: Pseudo-code for CE phase of MCEM^2^

1: ${\hat{\theta }}^{\left(0\right)}\leftarrow (1\text{,}1\text{,}\ldots \text{,}1)\text{,}\delta^{\left(0\right)}\leftarrow \infty \text{,}m\leftarrow 0$

2: **while**^*δ*(*m*)^>0**do**

3: m←m+1

4: $t_{0}\leftarrow 0$

5: $r_{\mathrm{jk}}^{\left(m\right)}\leftarrow 0\forall j,k$

6: **for***i*=1 to *d***do**

7: **for***k*=1 to *K***do**

8: generate ${\tilde{\theta }}_{i,k}^{\left(m-1\right)}\equiv \left({\tilde{\theta }}_{1,i,k}^{\left(m-1\right)},\ldots ,{\tilde{\theta }}_{M,i,k}^{\left(m-1\right)}\right)$ by evaluating Equation (8) *M* times

9: $t\leftarrow t_{i-1}$

10: **if***i*=1**then**

11: $x\leftarrow x_{0}$

12: **else**

13: x← final state of $z_{i-1,k}^{\left(m\right)}$

14: **end if**

15: **while***t*≤*t*_*i*_**do**

16: compute all *h*_*j*_(**x**)

17: generate *τ*,*j*^*′*^ using the SSA with ${\tilde{\theta }}_{i,k}^{\left(m-1\right)}$,augment $z_{i,k}^{\left(m\right)}$

18: t←t+τ, $r_{j^{\prime }k}^{\left(m\right)}\leftarrow r_{j^{\prime }k}^{\left(m\right)}+1$, update **x** to reflect the firing of reaction $R_{j^{\prime }}$

19: **end while**

20: **end for**

21: $\delta^{\left(m\right)}\leftarrow {\left(\rho \times 100\right)}^{\mathrm{th}}$ quantile of $\left(d(z_{i,1}^{\left(m\right)},y_{i}),\ldots ,\right)$$\left(d(z_{i,K}^{\left(m\right)},y_{i})\right)$

22: **if***i*<*d***then**

23: replace $\left(z_{i,1}^{\left(m\right)},\ldots ,z_{i,K}^{\left(m\right)}\right)$ by sampling with replacement from the $z_{i,k}^{\left(m\right)}$ satisfying $d(z_{i,k}^{\left(m\right)},y_{i})$≤*δ*^(*m*)^

24: **end if**

25: **end for**

26: compute ${\hat{\theta }}^{\left(m\right)}$ according to Equation (7)

27: **end while**

28: **return**${\hat{\theta }}^{\mathrm{CE}}={\hat{\theta }}^{\left(m\right)}$

## Algorithm 2: Pseudo-code for MCEM phase of MCEM^2^

1: ${\hat{\theta }}^{\left(0\right)}\leftarrow {\hat{\theta }}^{\mathrm{CE}}$, n←0

2: **while** (upper bound of the change in conditional log-likelihood > .005) **do**

3: n←n+1

4: **if***n*>1**then**

5: increment *K*^*′*^ as described in [19]

6: **end if**

7: $t_{0}\leftarrow 0$

8: $r_{jk^{\prime }}^{\left(n\right)}\leftarrow 0\forall j\text{,}k^{\prime }$

9: **for***i*=1 to *d***do**

10: **for***k*^*′*^=1 to *K*^*′*^**do**

11: $t\leftarrow t_{i-1}$

12: **if***i*=1**then**

13: $x\leftarrow x_{0}$

14: **else**

15: $x\leftarrow x_{i-1}^{\prime }$

16: **end if**

17: **while***t*≤*t*_*i*_**do**

18: compute all *h*_*j*_(**x**)

19: generate *τ*, *j*^*′*^ using the SSA with ${\hat{\theta }}^{\left(n-1\right)}$, augment $z_{i,k^{\prime }}^{\left(n\right)}$

20: t←t+τ, $r_{j^{\prime }k^{\prime }}^{\left(n\right)}\leftarrow r_{j^{\prime }k^{\prime }}^{\left(n\right)}+1$, update **x** to reflect the firing of reaction $R_{j^{\prime }}$

21: **end while**

22: **if**$d(z_{i,k^{\prime }}^{\left(n\right)},y_{i})>0$**then**

23: reset $z_{i,k^{\prime }}^{\left(n\right)}$, $r_{j^{\prime }k^{\prime }}^{\left(n\right)}$ to values held before step 17

24: **go to**step 11

25: **end if**

26: **end for**

27: **end for**

28: compute ${\hat{\theta }}^{\left(n\right)}$ according to Equation (5)

29: **end while**

30: **return**$\hat{\theta }={\hat{\theta }}^{\left(n\right)}$

## Algorithm 3: Pseudo-code for computing MCEM^2^ uncertainty estimates

1: $t_{0}\leftarrow 0$

2: $r_{jk^{\prime }}\leftarrow 0\forall j,k^{\prime }$

3: **for***i*=1 to *d***do**

4: **for***k*^*′*^=1 to *K*^*′*^**do**

5: $t\leftarrow t_{i-1}$

6: **if***i*=1**then**

7: $x\leftarrow x_{0}$

8: **else**

9: $x\leftarrow x_{i-1}^{\prime }$

10: **end if**

11: **while***t*≤*t*_*i*_**do**

12: compute all *h*_*j*_(**x**)

13: generate *τ*, *j*^*′*^ using the SSA with $\hat{\theta }$, augment ${zi\text{,}}_{k^{\prime }}$

14: t←t+τ, $r_{j^{\prime }k^{\prime }}\leftarrow r_{j^{\prime }k^{\prime }}+1$, update **x** to reflect the firing of reaction $R_{j^{\prime }}$

15: **end while**

16: **if***d*( ${zi,}_{k\prime }$,**y**_*i*_)>0**then**

17: reset $z_{i\text{,}k\prime }$, $r_{j\prime k\prime }$ to values held before step 11

18: **go to** step 5

19: **end if**

20: **end for**

21: **end for**

22: compute $\hat{\Sigma }$ according to Equation (10)

23: **return**$\hat{\Sigma }$

## Competing interests

The authors declare that they have no competing interests.

## Authors’ contributions

Conceived and designed the experiments: BJDJ MKR LRP JN. Performed the experiments: BJDJ MKR. Wrote the paper: BJDJ LRP JN. All authors read and approved the final manuscript.
